# Endogenous Secretory RAGE as a Novel Biomarker for Metabolic Syndrome and Cardiovascular Diseases

**Published:** 2007-09-17

**Authors:** Hidenori Koyama, Hiroshi Yamamoto, Yoshiki Nishizawa

**Affiliations:** 1 Department of Metabolism, Endocrinology and Molecular Medicine, Osaka City University Graduate School of Medicine, Osaka 545-8585, Japan; 2 Department of Biochemistry and Molecular Vascular Biology, Kanazawa University Graduate School of Medical Science, Kanazawa 920-8640, Japan

**Keywords:** receptor for advanced glycation end-products (RAGE), soluble RAGE (sRAGE), endogenous secretory RAGE (esRAGE), AGEs, atherosclerosis, metabolic syndrome, inflammation

## Abstract

Receptor for advanced glycation end-products (RAGE) is known to be involved in both micro- and macro-vascular complications in diabetes. Among numerous truncated forms of RAGE recently described, the C-terminally truncated form of RAGE has received much attention. This form of RAGE, carrying all of the extracellular domains but devoid of the transmembrane and intracytoplasmic domains, is released outside from cells, binds ligands including AGEs, and is capable of neutralizing RAGE signaling on endothelial cells in culture. This form of RAGE is generated as a splice variant and is named endogenous secretory RAGE (esRAGE). Adenoviral overexpression of esRAGE reverses diabetic impairment of vascular dysfunction, suggesting that esRAGE may be an important inhibitor of RAGE signaling in vivo and potentially be useful for prevention of diabetic vascular complications. An ELISA system to measure plasma esRAGE was recently developed, and the pathophysiological roles of esRAGE have begun to be unveiled clinically. Plasma esRAGE levels are decreased in patients with several metabolic diseases including type 1 and type 2 diabetes, metabolic syndrome and hypertension. In cross-sectional analysis, plasma esRAGE levels are inversely correlated with carotid or femoral atherosclerosis. In an observational cohort of patients with end-stage renal disease, cumulative incidence of cardiovascular death was significantly higher in subjects with lower plasma esRAGE levels. These findings suggest that plasma esRAGE may act as a protective factor against and a novel biomarker for the occurrence of metabolic syndrome and cardiovascular diseases.

## Receptor for Advanced Glycation End-products (RAGE) and its C-terminally Truncated Form (endogenous secretory RAGE, esRAGE)

RAGE is a multiligand cell-surface protein that was isolated from bovine lung in 1992 by the group of Schmidt and Stern.^1^ RAGE belongs to the immunoglobulin superfamily of cell-surface molecules, and binds to various ligands including AGEs, S100 (calgranulin), HMGB1 (amphoterin), and amyloid beta-peptides.^2^–^4^ Ligand engagement of RAGE in endothelial cells activates the transcription factor nuclear factor-κB (NF-κB), subsequently leading to increased expression of inflammatory mediators such as monocyte chemoattractant protein-1 (MCP-1) and vascular cell adhesion molecule-1 (VCAM-1).^5^,^6^ Accumulated evidence suggests that the receptor for advanced glycation end-products (RAGE) is involved in both diabetic micro-7–12 and macrovascular complications.^13^,^14^

**Figure 1:**
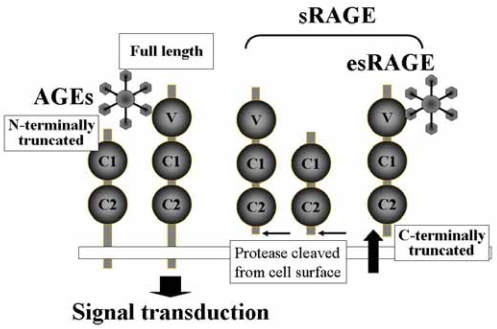
Numerous truncated forms of RAGE. There are three major spliced variants of RAGE: full length, N-terminally truncated, and C-terminally truncated. The C-terminally truncated form of RAGE is secreted from the cell and is named endogenously secreted RAGE (esRAGE). esRAGE has a V-domain, which is essential for binding with ligands, and is capable of competing with RAGE signaling as a decoy receptor. There are other forms of soluble RAGE (sRAGE) that are cleaved from cell-surface RAGE by matrix metalloproteinases. The ELISA assay for sRAGE measures all soluble forms including esRAGE in human plasma, while the ELISA for esRAGE measures only esRAGE, using polyclonal antibody raised against the unique C-terminus of the esRAGE sequence.

Numerous truncated forms of RAGE have recently been described^15^–^19^ (Fig. 1). Two major spliced variants of RAGE mRNA, N-terminal and C-terminal truncated forms, have been most extensively characterized.^16^ The N-truncated isoform of RAGE mRNA codes for a 303-amino-acid protein lacking the N-terminal signal sequence and the first V-like extracellular domain. The N-truncated form is incapable of binding with AGEs, since the V-domain is critical for binding of the ligand.^1^ The N-truncated form of RAGE appears to be expressed on the cell surface similar to the full-length RAGE, although its biological roles remain to be elucidated^4^. It has been suggested that this form of RAGE could be involved in angiogenic regulation in a fashion independent of the classical RAGE signaling pathway.^4^

The C-terminal truncated form of RAGE lacks the exon 10 sequences encoding the transmembrane and intracytoplasmic domains.^16^ This spliced variant mRNA of RAGE encodes a protein consisting of 347 amino acids with a 22-amino-acid signal sequence, and is released from cells. This C-truncated form is now known to be present in human circulation and is named endogenous secretory RAGE (esRAGE).^16^ esRAGE was found to be capable of neutralizing the effects of AGEs on endothelial cells in culture.^16^ Adenoviral overexpression of esRAGE in vivo in mice reverses diabetic impairment of vascular dysfunction.^20^ Thus, the decoy function of esRAGE may exhibit a feedback mechanism by which esRAGE prevents the activation of RAGE signaling. It has also been suggested that some soluble RAGE (sRAGE) isoforms that could act as decoy receptors may be cleaved proteolytically from the native RAGE expressed on the cell surface,^21^ suggesting heterogeneity of the origin and nature of sRAGE. This proteolytic generation of sRAGE was initially described as occurring in mice.^22^ The molecular heterogeneity of the diverse types of sRAGE in human plasma could exert significant protective effects against RAGE-mediated toxicity.

## sRAGE and esRAGE as Potential Biomarkers for Cardiovascular and Metabolic Diseases: Cross-sectional Clinical Studies (Table)

**Figure 2:**
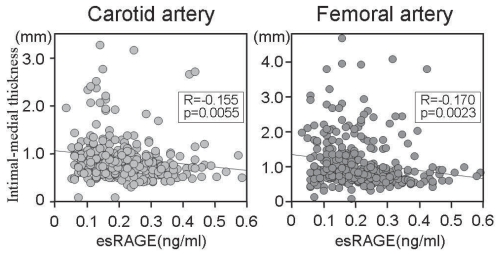
Plasma esRAGE level is inversely correated with carotid and femoral atherosclerosis. Atherosclerosis was determined as intimal-medial thickness measured by arterial ultrasound. N = 337 including 203 type 2 diabetic patients. Reproduced from a reference^26^.

Since sRAGE and esRAGE may be involved in feedback regulation of the toxic effects of RAGE-mediated signaling, recent clinical studies have focused on the potential significance of circulating sRAGE and esRAGE in a variety of pathophysiological conditions. First, Falcone et al.^23^ reported that total sRAGE levels are significantly lower in patients with angiographically proven coronary artery disease (CAD) than in age-matched healthy controls. The association between circulating sRAGE and angiographic observations was shown to be dose-dependent, with individuals in the lowest quartile of sRAGE exhibiting the highest risk for CAD. Importantly, this cohort consisted of a non-diabetic population, suggesting that the significance of sRAGE may not be confined to diabetes. Falcone et al also showed that the association between sRAGE and the risk of CAD was independent of other classical risk factors. The same research group also showed that patients with Alzheimer disease have lower levels of sRAGE in plasma than patients with vascular dementia and controls, suggesting a role for the RAGE axis in this clinical entity as well.^24^ Following development of an ELISA system to specifically measure human esRAGE,^25^ we measured plasma esRAGE level and cross-sectionally examined its association with atherosclerosis in 203 type 2 diabetic and 134 non-diabetic age- and gender-matched subjects.^26^ In this study, type 2 diabetes was diagnosed by fasting plasma glucose >126 mg/dl (7 mmol/L), causal plasma glucose >200 mg/dl (11.1 mmol/L), or 2-hour plasma glucose >200 mg/dl during 75 g oral glucose tolerance test, or previous treatment for diabetes. esRAGE levels were inversely correlated with carotid and femoral atherosclerosis, as measured as intimal-medial thickness (IMT) by arterial ultrasound (Fig. 2). Stepwise regression analyses revealed that plasma esRAGE was the third strongest and an independent factor associated with carotid IMT, following age and systolic blood pressure. Another Japanese research group also found an inverse correlation between plasma esRAGE and carotid atherosclerosis in type 1^27^ and type 2 diabetic subjects.^28^ Thus, plasma esRAGE or sRAGE may protect against the occurrence of cardiovascular diseases, though this hypothesis needs to be tested in a longitudinal cohort study.

Several metabolic components well-established as risk factors for cardiovascular diseases have also been shown to be associated with altered plasma sRAGE or esRAGE levels. We have shown that plasma esRAGE levels are decreased in subjects with metabolic syndrome and are inversely correlated with several components of metabolic syndrome including body mass index, blood pressures, insulin resistance index, fasting plasma glucose, serum triglyceride, and lower HDL-cholesterol levels.^26^ The majorities of these correlations remained significant even when the non-diabetic or type 2 diabetic subpopulation was extracted for analyses, again suggesting that plasma esRAGE plays important roles even in the non-diabetic population. An inverse correaltion between esRAGE (or sRAGE) and body mass index was also found for control subjects,^29^ those with type 1 diabetes,^30^ and those with end-stage renal disease (ESRD).^31^ Moreover, patients with hypertension have been found to have lower plasma sRAGE or esRAGE levels.^26^,^32^

The findings regarding plasma levels of the soluble form of RAGE in diabetes are quite confusing. We and other groups have found that plasma esRAGE level is significantly lower in type 1 and type 2 diabetic patients than in non-diabetic controls.^26^,^27^ However, plasma sRAGE levels have been shown to be increased in type 1^33^ and type 2 diabetic patients,^34^,^35^ although conflicting findings have been reported.^36^ Of note, when diabetic subjects alone were extracted for analyses, a direct association was not observed between plasma soluble RAGE (both sRAGE and esRAGE) levels and the status of glycemic control (ie, glycohemoglobin A1c).^26^,^30^,^36^–^38^ Thus, these complex findings in diabetic subjects suggest that levels of plasma soluble forms of RAGE are not determined simply by status of glycemic control, and that even plasma esRAGE and sRAGE levels may be under the control of distinct mechanisms.

## Plasma esRAGE Levels in Chronic Kidney Diseases

**Figure 3:**
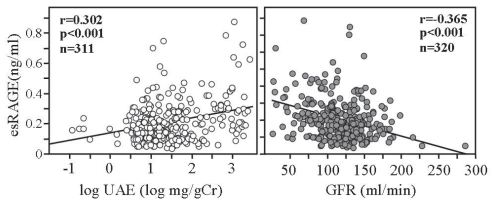
Plasma esRAGE level is influenced by the presence of kidney disease in patients with type 2 diabetes. UAE: urinary albumin excretion, GFR: glomerular filtration rate estimated by MDRD equation.

Abnormal chronic inflammation associated with progressive, chronic kidney disease (CKD) reflects sustained activation of inflammatory cells, such as monocytes/macrophages, in which accumulation of AGEs may play an important role through binding with RAGE. It has been shown that, in peripheral monocytes from subjects with varying severities of CKD, RAGE expression is closely associated with worsening of CKD and is strongly correlated with plasma levels of pentosidine, a marker for AGEs.^39^ In ESRD subjects with high-grade inflammation, stimulation of mononuclear cells with AGE-modified human serum albumin causes a rapid, dose-dependent increase in NF-κB activity that could be completely blocked by an anti-RAGE antibody.^40^ Thus, enhanced RAGE expression in ESRD may amplify AGEs-induced perturbation and contribute to systemic inflammatory diseases like atherosclerosis. Circulating esRAGE and sRAGE levels have been shown to be increased in patients with decreased renal function, particularly those with ESRD.^34^,^38^,^41^ As shown in Figure 3, plasma esRAGE levels are positively correlated with urinary excretion rate, and inversely correlated with estimated glomerular fitration rate in type 2 diabetic patients. It remains to be determined whether this increase is caused by decreased renal function alone or whether esRAGE levels are upregulated to protect against toxic effects of the RAGE ligands. Successful kidney transplantation resulted in significant decrease in plasma sRAGE,^42^ implying that the kidneys play a role in sRAGE removal.

## Low circulating esRAGE Level predicts Cardiovascular Diseases: A Longitudinal Study

We recently reported an observational cohort study in patients with ESRD and for the first time longitudinally evaluated the effect of plasma esRAGE on cardiovascular mortality.^31^ Patients with ESRD have been reported to have a substantially increased rate of cardiovascular mortality. The cohort in that study included 206 ESRD subjects registered between June 1992 and June 1995, who had been treated by regular hemodialysis for more than 3 months. At baseline, plasma esRAGE levels in ESRD patients were higher than those in subjects without renal disease. Similar to subjects without renal disease, diabetic ESRD subjects exhibited significantly lower plasma esRAGE levels than non-diabetic ESRD subjects. Plasma esRAGE levels were inversely correlated with many of the components of metabolic syndrome, such as increase in body mass index, increase in fasting plasma glucose, increased triglyceride level and decreased HDL cholesterol, even in ESRD subjects. The subjects were followed up until December 2001, with a median follow-up period of 111 months. At the end of follow-up, 132 patients were confirmed to be alive on hemodialysis and 74 to have died. The 74 deaths during follow-up included 34 due to fatal cardiovascular events.

**Figure 4:**
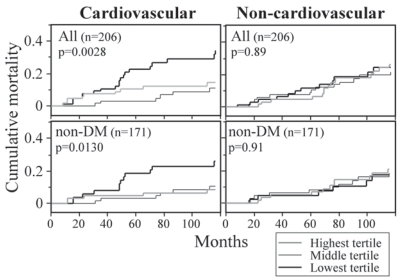
Low plasma esRAGE level is a predictor of cardiovascular mortality in patients with ESRD. Cumulative mortalities in subjects with the lowest, middle, and highest tertiles of plasma esRAGE levels were estimated by Kaplan Meier analysis and the log-rank test. Reproduced from a reference.^31^

Even though the plasma esRAGE levels at baseline were higher in ESRD subjects than in those without kidney disease, the subjects in the lowest tertile of plasma esRAGE levels exhibited significantly higher cardiovascular mortality, but not non-cardiovascular mortality (Fig. 4). Univariate Cox proportional hazards analyses revealed that, compared with the subjects in the lowest tertile of plasma esRAGE levels, those in the middle and highest tertiles had significantly less risk of cardiovascular mortality (hazard ratios 0.26 and 0.40, respectively). Multivariate Cox proportional hazards analyses revealed that the higher risk of the subjects with lower esRAGE levels was not significant when adjusted for age, fasting plasma glucose or presence of diabetes, suggesting that esRAGE, aging, and glycemic control mutually interact in the regulation of cardiovascular mortality. Adjustment for other confounders of esRAGE (body mass index, triglyceride, and HDL cholesterol) barely affected the significant association between lower esRAGE level and cardiovascular mortality. Moreover, the association of lower esRAGE level with cardiovascular mortality was independent of other predictors of cardiovascular mortality such as serum creatinine, non-HDL cholesterol, HbA1c, and vascular complications. Our findings thus suggest that low circulating esRAGE level is a predictor for atherosclerosis and cardiovascular events in patients with ESRD. Since this study was designed to survey predictors for cardiovascular mortality in the population of ESRD patients, and the number of fatal events was relatively small and statistical power may not have been high enough to detect important cardiovascular risk factors, demonstration of the role of plasma esRAGE as a biomarker of cardiovascular mortality will require further large-scale prospective studies or nested case-control studies with required numbers of subjects calculated by power analysis.

It is not known at present how esRAGE is involved in cardiovascular mortality. In our ESRD cohort, neither plasma pentosidine nor carboxy-methyl-lysine level predicted cardiovascular mortality. Moreover, the inverse correlation between low circulating esRAGE level and cardiovascular mortality was not dependent of plasma AGEs levels. Thus, the protective effect of esRAGE against cardiovascular mortality may not be entirely dependent on neutralization of toxic AGEs. Other endogenous ligands for RAGE, such as S100A12, may also be involved in the function of esRAGE. The plasma level of S100A12 has been shown to be increased in diabetes and inversely correlated with serum sRAGE level.^36^,^43^

## sRAGE vs. esRAGE: Distinct Pathophysiological Significances?

It is unclear at present whether the pathophysiological significances of circulating esRAGE and sRAGE are distinct in different clinical settings. It appears that esRAGE represents less than half of the total sRAGE in human plasma. In our analyses, plasma esRAGE level in Japanese healthy subjects was found to be 0.25 ± 0.11 ng/ml,^26^ while mean plasma sRAGE level in Caucasian healthy controls has been reported to be 1.3 ng/ml.^23^ We and others have shown that plasma esRAGE level is decreased in diabetes.^26^,^27^ In contrast to the case of esRAGE, circulating sRAGE levels are increased, rather than decreased, in both type 1 and type 2 diabetic patients,^33^–^35^ with one conflicting report.^36^ Humpert et al also showed that sRAGE but not esRAGE is associated with albuminuria in patients with type 2 diabetes.^38^ Yamamoto et al^44^ recently described a head-to-head comparison of plasma esRAGE and sRAGE levels using esRAGE as a standard protein and different sets of antibodies, and showed that plasma esRAGE level was about 2-fold less than that of plasma sRAGE. In their analysis, esRAGE and sRAGE levels were positively correlated, with a stronger correlation in healthy subjects than in type 1 diabetic patients. Thus, the possibility of distinct roles for them in certain disease conditions requires further examination.

## Soluble RAGE as a Therapeutic Target?

The potential usefulness of soluble RAGE for prevention and treatment of inflammatory diseases has been demonstrated in many animal models. Blockade of RAGE by administration of genetically engineereds RAGE successfully prevented the development of micro-8,9 and macrovascular complications in diabetes.^45^–^47^ We have also shown that adenoviral over-expression of esRAGE successfully restored the impaired angiogenic response in diabetic mice.^20^ Sakaguchi et al found that administration of sRAGE markedly suppressed neointimal formation following arterial injury in non-diabetic mice.^48^ Soluble RAGE has also been shown to effectively prevent the development of diabetes,^49^ protect against tumor growth and metastasis,^50^ improve the outcome of colitis,^51^ restore impaired wound healing,^52^ and suppress Alzheimer disease-like conditions.^53^ These effects of soluble RAGE in animal models could be explained by its decoy function, inhibiting RAGE interaction with its proinflammatory ligands, which might be applicable to human diseases as well.

Further application of soluble RAGE to the treatment of human diseases will require answers to several questions. Most importantly, limited findings are available regarding the mechanisms of regulation of circulating esRAGE or sRAGE in humans. A tissue microarray technique using a wide variety of adult normal human preparations obtained from surgical and autopsy specimens revealed that esRAGE was widely distributed in tissues, including vascular endothelium, monocyte/macrophage, pneumocytes, and several endocrine organs.^54^ However, it is unclear at present from which organ or tissue plasma sRAGE or esRAGE originate. Circulating AGEs may be involved in regulation of the secretion or production of soluble RAGE, since AGEs are known to upregulate RAGE expression in vitro.^55^ esRAGE could be simultaneously upregulated by AGEs and act as a negative feedback loop to compensate for the damaging effects of AGEs. Several studies have found positive correlations between plasma sRAGE or esRAGE and AGEs. 29–31,34 This possibility is further supported by the findings that the suppression of sRAGE expression in diabetic rat kidney is reversed by blockade of AGEs accumulation with alagebrium.^56^ Other inflammatory mediators, such as S100, tumor necrosis factor α, and C-reactive protein, could also be potential candidates for regulation of the plasma level of soluble RAGE in humans.^36^,^55^,^57^ Without doubt, further understanding of the regulation of soluble RAGE will be most helpful in delineating potential targets for therapeutic application of soluble RAGE.

Second, it would be important to determine whether currently available pharmacological agents can regulate plasma sRAGE or esRAGE. Forbes et al^58^ showed that inhibition of angioten-sin-converting enzyme (ACE) in rats increased renal expression of sRAGE, and that this was associated with decreases in expression of renal full-length RAGE protein. They also showed that plasma sRAGE levels were significantly increased by inhibition of ACE in both diabetic rats and in human subjects with type 1 diabetes. Thus, one attractive scenario is that the protective effect of ACE inhibition against progression of renal dysfunction is mediated through regulation of RAGE versus soluble RAGE production. Other potential agents that may affect circulating soluble RAGE include the thiazolidinediones^59^ and statins^60^,^61^, both of which are known to modulate the AGEs-RAGE system in culture, although their effects on secretion of soluble RAGE are not known.

Taken altogether, the findings discussed here suggest that sRAGE or esRAGE could serve as a novel biomarker for estimation of the risk of progression of atherosclerotic disorders. Further examination of the molecular mechanisms underlying RAGE and esRAGE regulation will provide important insights into potential targets for the prevention and treatment of cardiovascular diseases.

## Figures and Tables

**Table:** Levels of Circulating soluble RAGE in cardiovascular and metabolic diseases.

SRAGE		references
CAD (non-DM)	decreased	23
	increased	35
Diabetes (type 1)	increased	33
Diabetes (type 2)	increased	34,35
	decreased	36
Hypertension	decreased	32
Alzheimer’s disease	decreased	24
**EsRAGE**		
Metabolic syndrome	decreased	26
Diabetes (type 1)	decreased	25,27
Diabetes (type 2)	decreased	26
Hypertension	decreased	26
Atherosclerosis (IMT)	inverse relation	26–28
